# Advanced feature selection and temporal attention mechanisms with Bi-LSTM classifier for optimizing emotion recognition in Kashmiri speech

**DOI:** 10.3389/frai.2026.1768701

**Published:** 2026-03-18

**Authors:** GH Mohmad Dar, Radhakrishnan Delhibabu

**Affiliations:** 1 School of Advance Sciences, Vellore Institute of Technology, Vellore, Tamil Nadu, India; 2 School of Computer Science and Engineering, Vellore Institute of Technology, Vellore, Tamil Nadu, India

**Keywords:** Attention mechanism, Harmonic to Noise Ratio (HNR), Kashmiri language, linear predictive coding (LPC), long short-term memory (LSTM), Mel-frequency cepstral coefficients (MFCC)

## Abstract

This study introduces an advanced methodology for enhancing emotion recognition in Kashmiri speech by leveraging optimized feature selection and integrating temporal attention mechanisms into Long Short-Term Memory (LSTM) networks. A meticulous feature selection process identified key acoustic features, including Mel Frequency Cepstral Coefficients (MFCCs), Linear Predictive Coding (LPC), and other relevant descriptors, as optimal for emotion classification. The incorporation of temporal attention layers significantly improved the model's capacity to capture complex emotional patterns and temporal dynamics within the speech data. The proposed attention-augmented LSTM model achieved an accuracy of 90.2%, outperforming the baseline LSTM model's accuracy of 86%. Notable improvements in precision, recall, and F1-scores across multiple emotional categories further highlight the efficacy of the attention mechanism in capturing subtle emotional variations. In addition to performance gains, the study provides a clear research direction by demonstrating how attention–based temporal modeling can benefit low-resource languages such as Kashmiri, where linguistic and prosodic cues differ significantly from widely studied languages. The findings therefore establish a methodological baseline that supports future SER deployments in digital domains, including chat-based systems, affect-aware agents, and other human–machine interfaces. These findings underscore the model's ability to enhance both the sensitivity and specificity of emotion recognition systems, offering a robust and efficient framework for speech-based emotion analysis. Future work will extend the proposed methodology to multilingual settings and incorporate multimodal information, enabling deeper analysis of emotional expression across diverse linguistic and cultural contexts.

## 1 Introduction

Speech Emotion Recognition (SER) represents a crucial domain within affective computing, enabling machines to interpret human emotions from vocal signals (Dar and Delhibabu, 2024c,b,a). Developing robust SER systems holds transformative potential across various fields, including mental health diagnostics, human-computer interaction, and automated customer support (Hashem et al., 2023). Despite significant advancements in SER for well-researched languages such as English and Mandarin, there remains a notable gap for underrepresented languages. The Kashmiri language, predominantly spoken in the Kashmir Valley, exemplifies this gap; prior to this work, no annotated datasets or established SER methodologies existed for Kashmiri. This study bridges this gap by developing the first comprehensive Kashmiri Emotional Speech Corpus (KESC) and evaluating the effectiveness of various acoustic feature combinations and temporal modeling architectures. Beyond linguistic interest, developing SER systems for low-resource languages carries tangible real-world importance. Emotional cues in speech play a vital role in human communication and directly support applications in clinical affect monitoring, intelligent tutoring systems, call-center analytics, assistive technologies, crisis intervention platforms, and human–robot interaction. The absence of such tools for Kashmiri not only restricts the advancement of culturally aligned AI systems but also constrains the digital visibility and technological modernization of the language.

Also emotion recognition in speech is inherently challenging due to the complex and dynamic nature of acoustic signals (Schuller et al., 2009). While Mel-frequency cepstral coefficients (MFCCs) have been widely used to capture spectral characteristics of speech (Majeed et al., 2015), recent research suggests that combining MFCCs with additional descriptors—such as Linear Predictive Coding (LPC), spectral features, chroma, formant frequencies, and harmonic-to-noise ratio (HNR)—provides a more comprehensive representation of emotional cues (Teixeira et al., 2023). Thus emotional expression in speech is not only an acoustic phenomenon but also a deeply linguistic and cultural one. Languages differ in how they shape prosodic contours, intonation patterns, vowel articulation, and temporal dynamics, and these differences influence how emotions are conveyed and perceived. Because of this diversity, insights and feature heuristics derived from high-resource languages, such as English or Mandarin, cannot be assumed to generalize to low-resource linguistic contexts. This disconnect creates a compelling need to investigate how emotional cues are encoded in Kashmiri speech and to determine which acoustic descriptors most effectively capture those nuances. Moreover bridging this gap extends beyond academic interest. In the absence of resources for speech and emotion processing, Kashmiri risks exclusion from emerging speech-driven technologies and digital services. By establishing foundational datasets and modeling benchmarks, this work advances SER research and brings necessary digital attention to Kashmiri, helping maintain its visibility and functional relevance in contemporary AI systems. Such benchmarks also provide a basis for future research and support the integration of Kashmiri into broader computational and technological ecosystems. We employ Bidirectional Long Short-Term Memory (Bi-LSTM) networks, which are well-suited to capture the sequential dependencies inherent in speech signals (Van Houdt et al., 2020). By processing sequences in both forward and backward directions, Bi-LSTM networks are able to capture context from the entire utterance, enhancing the detection of subtle emotional nuances. Beyond the baseline Bi-LSTM, we evaluate the different feature combination configuration across additional architectures, including Gated Recurrent Units (GRU), Temporal Convolutional Networks (TCN), and Deep Convolutional Neural Networks (DCNN) and also test it on up-to-mark datasets from languages Urdu, Persian, and English to validate the generalizability of our approach. To ensure methodological rigor, we perform comprehensive feature evaluation and selection using quantitative and statistical measures, including ROC curve analysis, box plots, and significance testing (ANOVA and paired *t*-tests). This allows us to identify the most discriminative and non-redundant feature set, which, combined with optimized temporal models, yields a robust framework for Kashmiri SER. This work represents the first systematic study for SER in Kashmiri, providing both a benchmark dataset and a validated methodological framework for advanced features selection for Kashmiri language. By combining feature optimization with advanced temporal modeling and cross-architecture validation, this study not only advances SER for Kashmiri but also offers insights applicable to other low-resource languages.

### 1.1 Research gaps and motivation

The motivation for this study and the key research gaps in Kashmiri SER can be summarized as follows:

- **Digital relevance:** Despite being an official language of India, Kashmiri remains largely unexplored in computational research. The lack of SER resources limits its integration into chatbots, virtual assistants, and other speech-driven technologies.
- **Language-specific characteristics:** Emotional expression in Kashmiri is shaped by its unique prosody, pitch, and temporal patterns, which differ from well-studied languages and influence the effectiveness of acoustic features for SER.
- **Feature and temporal analysis:** The optimal combination of MFCCs and complementary acoustic features for Kashmiri speech remains unexplored. Additionally, systematic evaluation of these features across temporal models such as Bi-LSTM, GRU, TCN, and DCNN has not been conducted, leaving gaps in understanding both feature effectiveness and sequence modeling for this language.
- **Benchmark need:** Creating a curated dataset and evaluating models establishes a foundation for future research and integration into digital applications.

## 2 Related study

Speech Emotion Recognition (SER) has become an important area in Human-Computer Interaction (HCI), with many studies focusing on how to detect emotions from speech. This research covers various steps, including collecting emotional speech data, applying preprocessing and feature extraction methods, and using different classifiers for emotion detection. In this section, we review the existing work in these areas, with special focus on SER in low-resource and unexplored languages. We first discuss the development of datasets, followed by techniques used for preprocessing and feature selection. Lastly, we review classification methods, ranging from basic machine learning models to advanced deep learning approaches.

### 2.1 SER in under-represented languages

Efforts toward emotion recognition in regional Indian languages have grown in recent years. For example, the IITKGP-SEHSC database for Hindi (Koolagudi et al., 2011), SUBESCO for Bangla (Mohanty and Swain, 2011), and datasets for Urdu, Marathi, and Malayalam (Asghar et al., 2022; Geethashree and Ravi, 2017; Jacob, 2017) demonstrate increasing focus on linguistic diversity. However, the scarcity of annotated corpora continues to hinder progress, motivating research into efficient data representation and temporal modeling strategies.

Traditional approaches have primarily relied on classifiers such as Support Vector Machines (SVMs), Gaussian Mixture Models (GMMs), and Random Forests combined with spectral features like MFCCs, LPCs, and pitch (Kamaruddin et al., 2014; Lalitha et al., 2019; Teixeira et al., 2022). While effective for smaller datasets, such models fail to capture the temporal dynamics of emotion-laden speech. The introduction of deep learning architectures such as Convolutional Neural Networks (CNNs) and Long Short-Term Memory (LSTM) networks has markedly advanced SER, particularly for modeling both spatial and temporal dependencies (Christy et al., 2020; Yang et al., 2016; Issa et al., 2020). These results suggest that deep learning methods, combined with good quality data, can significantly improve SER performance in low-resource languages. Table 1 summarizes some works on speech emotion recognition for Indo-Aryan and Dravidian languages.

**Table 1:** Summary of SER studies in Indo-Aryan and Dravidian languages.

**S. No**.	**References**	**Dataset**	**Language(s)**	**Type**	**Emotions covered**
1	(Syed et al. 2020)	Urdu-Sindhi speech emotion corpus	Urdu, Sindhi	Audio	Happiness, anger, sadness, disgust, surprise, sarcasm, and neutral
2	(Samantaray et al. 2015)	MESDNEI	Assamese	Audio	Happy, anger, fear, disgust, surprise, sad, and neutral
3	(Ali et al. 2015)	Speech emotional corpus	Urdu, Sindhi, Pashto, Punjabi, Balochi	Audio	Happiness, sad, anger, and neutral
4	(Darekar and Dhande 2018)	Marathi database	Marathi	Audio	Happy, sad, angry, fear, neutral, and surprised
5	(Basu and Majumder 2020)	Santali Speech Data	Santali	Audio	Anger, fear, happy, sad, surprise, and neutral
6	(Dhar and Guha 2021)	Bangla emotional speech dataset	Bangla	Audio	Happy, angry, and neutral
7	(Fernandes and Mannepalli 2021)	Hindi emotional speech database	Hindi	Audio	Happy, sad, anger, fear, surprise, disgust, and neutral
8	(Tank and Hadia 2020)	Gujarati speech database	Gujarati	Audio	Sadness, surprise, anger, disgust, fear, and happiness
9	(Aziz et al. 2023)	BanglaSER	Bengali	Audio	Anger, disgust, happiness, sadness, surprise, fear, and neutral

### 2.2 Preprocessing and feature extraction in SER

Preprocessing and feature extraction are pivotal to SER performance, as they determine the discriminative quality of acoustic features. Common preprocessing operations include silence removal, pre-emphasis, normalization, and noise reduction (Sefara, 2019; Harár et al., 2017; Sajjad et al., 2020). These operations ensure that emotionally salient portions of speech dominate the analysis. However, end-to-end deep models that process spectrograms or mel-spectrograms often minimize preprocessing to preserve subtle cues (Lee et al., 2020).

Feature extraction techniques encompass prosodic, spectral, and voice-quality domains. Among these, Mel-Frequency Cepstral Coefficients (MFCCs) are most popular due to their perceptual alignment with the human auditory system (Davis and Mermelstein, 1980; Li et al., 2014). Studies indicate that combining MFCCs with Chroma, LPC, Pitch, Energy, and Spectral Contrast enhances robustness (Lalitha and Tripathi, 2016; Zhou et al., 2010; Pan et al., 2005; Tellai et al., 2024). Recent work has shown that SER performance can improve significantly when MFCCs are combined with complementary acoustic descriptors, rather than being used in isolation (Hassan et al., 2024). Table 2 summarizes commonly adopted preprocessing and feature extraction strategies in recent SER literature.

**Table 2:** Preprocessing techniques and features used in recent SER studies.

**S. No**.	**References**	**Preprocessing techniques**	**Features used**
1	(Mohanty and Cherukuri 2024)	Z-normalization, silence removal	MFCC, spectral entropy, chroma features
2	(Chowdhury et al. 2025)	Pre-emphasis, noise reduction	MFCC, ZCR, Chroma, STFT
3	(Leem et al. 2023)	Min-max normalization, segmentation	Acoustic Features
4	(Yuanchao et al. 2023)	Data augmentation (pitch shifting), normalization	Mel-spectrogram, pitch, intensity
5	(Kaur and Singh 2022)	Noise addition, time warping	MFCC, spectral contrast, tonnetz
6	Sajjad et al. (2020)	K-means clustering, STFT	Spectrogram-based CNN features

### 2.3 Deep learning and temporal modeling approaches

Deep learning has become the dominant paradigm in SER, enabling models to learn complex temporal dependencies from acoustic signals. LSTM networks (Hochreiter and Schmidhuber, 1997) and their bidirectional variants (Bi-LSTMs) (Joshi et al., 2022) are particularly effective in modeling emotion trajectories across time. CNN-LSTM hybrids combine spatial feature extraction and temporal sequence modeling, yielding higher accuracy and robustness (Issa et al., 2020). Temporal Convolutional Networks (TCNs) (Bai et al., 2018) and Gated Recurrent Units (GRUs) (Cho et al., 2014; Tellai and Mao, 2023) provide computationally efficient alternatives with strong generalization ability. Additionally, attention-based LSTM architectures enhance interpretability by assigning dynamic importance to emotionally relevant frames (Khan et al., 2024; Tursunov et al., 2021).

Table 3 presents representative deep learning models used in SER research and their reported performance trends.

**Table 3:** Recent deep learning classifiers used in SER studies.

**S. No**.	**References**	**Model architecture**	**Key highlights**	**Reported accuracy (%)**
1	(Issa et al. 2020)	CNN-LSTM hybrid	Combines CNN spatial features with LSTM temporal modeling on RAVDESS dataset	86.5
2	(Yang et al. 2016)	Deep CNN	Learns hierarchical emotional features from spectrograms	83.2
3	(Joshi et al. 2022)	Bi-LSTM	Models long-term temporal dependencies effectively; tested on EMO-DB	88.0
4	(Khan et al. 2024)	Attention-based Bi-LSTM	Incorporates attention to focus on emotionally salient frames	89.3
5	(Bai et al. 2018)	Temporal Convolutional Network (TCN)	Uses dilated convolutions for efficient sequence modeling	85.1
6	(Manohar and Logashanmugam 2022)	CNN-GRU hybrid	Integrates CNN front-end with GRU layers for reduced complexity	84.7
7	(Wang et al. 2023)	Federated TCN	Distributed learning for privacy-preserving SER applications	83.9
8	(Christy et al. 2020)	Multimodal LSTM	Fusion of audio and visual cues for emotion recognition	90.2
9	(Tellai et al. 2023)	CNN-transformer fusion network	lightweight speech emotion recognition system	97.64%, 99.42%, and 97.53%

### 2.4 Aims and contributions

This study is motivated by the absence of Speech Emotion Recognition (SER) research for the Kashmiri language and addresses a dual scientific and practical gap: (i) the lack of annotated emotional speech resources and (ii) the lack of empirical benchmarks for feature selection and temporal modeling in low-resource settings The key aims and novel contributions are as follows:

- **Creation of the first annotated Kashmiri Speech Emotion Corpus (KESC):** We introduce a high-quality, fully annotated audio dataset in Kashmiri, covering multiple emotional categories. This dataset overcomes challenges such as the lack of publicly available text and audio data and establishes the first benchmark for SER in Kashmiri, providing a vital resource for future research and language preservation.
- **Comprehensive feature optimization for low-resource language SER:** We systematically evaluate multiple acoustic feature combinations including MFCC, LPC, spectral features, chroma, formants, and HNR to identify the most discriminative feature set. This ensures accurate and efficient emotion recognition while offering insights into which features are most informative for Kashmiri speech, a previously unexplored area.
- **Temporal modeling and cross-architecture validation:** The optimal feature configuration is validated across several deep learning architectures Bi-LSTM, GRU, TCN, and DCNN to ensure robustness and generalizability. This comprehensive evaluation provides actionable insights into temporal modeling strategies for low-resource languages.
- **Establishment of a benchmark SER framework for Kashmiri:** By combining optimized features with attention-enhanced temporal models, we deliver a robust SER framework achieving high accuracy, balanced class performance, and reliable generalization. This framework not only provides a benchmark for Kashmiri but also lays the foundation for future SER research in other underrepresented languages, contributing to language digitization and preservation efforts.

## 3 Methodology

This study presents a systematic methodology aimed at optimizing emotion recognition in Kashmiri speech through advanced feature selection and temporal attention mechanisms. The main objective is to analyze how different combinations of acoustic features influence emotional representation and classification accuracy, and to determine the most effective feature set for reliable recognition. The methodology follows a two-stage evaluation framework. In the first stage, a Bi-LSTM network is used as the baseline classifier to identify the optimal combination of acoustic features that maximizes emotional discriminability in Kashmiri speech. In the second stage, the identified feature set is validated using additional deep learning architectures such as GRU, DCNN, and TCN to evaluate its generalization ability across models. This multi-model validation ensures that the selected features are not dependent on any single architecture but instead capture the intrinsic emotional patterns embedded in the Kashmiri acoustic domain.

**Figure 1:**
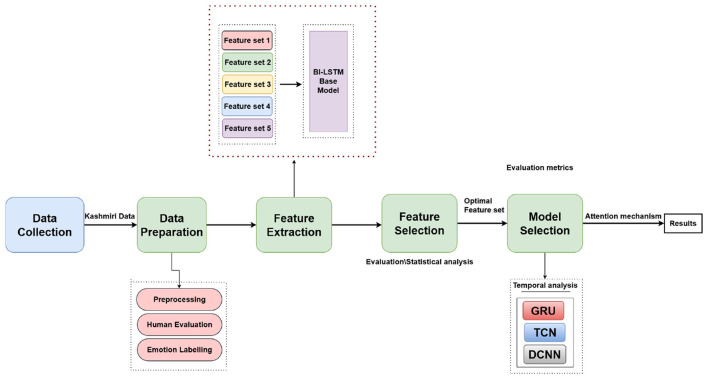
An overview of the methodology process.

The process begins with the preparation of a clean and balanced speech corpus, ensuring that all recordings are free from background noise and represent genuine emotional expressions. A diverse set of acoustic features including Mel-Frequency Cepstral Coefficients (MFCC), Linear Predictive Coding (LPC), spectral, chroma, and other prosodic features are extracted and analyzed to examine their discriminative capability. Temporal attention modeling is further applied to capture how emotional cues evolve over time, providing a more detailed understanding of the dynamic nature of emotions in Kashmiri speech. The subsequent subsections describe the stages of data preparation, feature extraction and selection, and experimental setup used in this study. Figure 1 presents an overview of the complete methodological framework.

### 3.1 Data preparation and pre-processing

The absence of annotated emotional speech corpora for Kashmiri presents a key barrier to advancing SER in this language. To address this, we designed a rigorous data collection and annotation framework that emphasizes linguistic authenticity, demographic diversity, and high-quality labeling.

#### 3.1.1 Data collection

The corpus was created from 166 native Kashmiri speakers (88 male, 78 female) representing different age groups and regional dialects. Participants were drawn from northern, southern, and central regions of Kashmir to capture dialectal variation and ensure generalizability. The final dataset spans approximately 5 h and 30 min of speech.

To maintain realism, 80% of the data consists of natural emotional speech, collected through semi-structured dialogues and storytelling tasks that elicited spontaneous emotions such as anger, happiness, sadness, and excitement. Controlled recordings (10%) were also included, where participants read phonetically balanced sentences designed by linguists and cultural experts. These acted samples ensured balance across emotion classes. The remaining 10% was sourced from ethically permissible public material, such as interviews, podcasts, and YouTube content, providing diversity in speaking styles, environments, and emotional intensities.

#### 3.1.2 Recording setup and quality control

All recordings were captured using studio-grade condenser microphones at 16 kHz, 16-bit resolution, and stored in mono-channel WAV format. Controlled sessions were held in acoustically treated environments, following a standardized protocol for microphone placement, noise control, and calibration. This ensured uniformity across sessions and minimized channel variability, producing clean, and high-quality speech essential for robust SER.

#### 3.1.3 Recording setup and quality control

All recordings were captured using studio-grade condenser microphones at 16 kHz, 16-bit resolution, and stored in mono-channel WAV format. Controlled sessions were held in acoustically treated environments, following a standardized protocol for microphone placement (approximately 15–20 cm from the speaker's mouth at a slight angle to reduce plosive artifacts), noise control, and calibration. This ensured uniformity across sessions and minimized channel variability, producing clean, high-quality speech essential for robust SER. Publicly sourced recordings (10% of the dataset) were carefully curated to minimize excessive background noise or reverberation, ensuring consistency with studio recordings.

#### 3.1.4 Raw audio pre-processing pipeline

The collected audio underwent a structured preprocessing pipeline to standardize quality. Noise reduction was applied using Audacity tools, including adaptive filtering and spectral subtraction. Peak normalization ensured consistent loudness across all samples, preventing volume variations from affecting model performance. Silence trimming removed non-speech intervals. All recordings were saved as 16 kHz, 16-bit mono WAV files, ensuring compatibility and reproducibility for feature extraction tasks such as MFCCs and spectrograms. This pipeline was uniformly applied across Kashmiri to maintain consistency for cross-lingual experiments.

#### 3.1.5 Human evaluation and labeling

Emotion annotation was conducted through a carefully structured two-stage human evaluation process designed to minimize subjectivity and ensure linguistic and cultural fidelity. The evaluators consisted of four native Kashmiri speakers with formal expertise in Kashmiri linguistics and phonetics. Each rater possessed prior experience in linguistic annotation and prosodic analysis, ensuring a high level of perceptual and analytical accuracy. Their socio-cultural and dialectal diversity further provided balanced representation across different variants of the Kashmiri language.

The annotation process comprised the following stages:

1. **Stage 1—Independent annotation:** Each rater independently annotated the audio samples using a Google Form interface. They were instructed to:

(a) Identify the dominant emotion expressed in the utterance.
(b) Justify their selection by referencing prosodic cues such as pitch, tone, rhythm, and intensity.
(c) Rate the emotional intensity on a five-point Likert scale (1–5).

Labels were initially assigned through majority voting where inter-rater agreement was strong.

**2. Stage 2—Expert adjudication:** In cases of disagreement particularly for acoustically or semantically overlapping emotions such as *happiness* vs. *excitement*an adjudication panel comprising Kashmiri linguists and speech emotion recognition (SER) experts reviewed the contested samples. Final labels were determined through consensus, ensuring perceptual validity, linguistic consistency, and cultural appropriateness.

This two tier evaluation framework ensured that the final dataset authentically represents emotional expressions within the Kashmiri linguistic context, balancing both objective analysis and native perceptual understanding.

**Figure 2:**
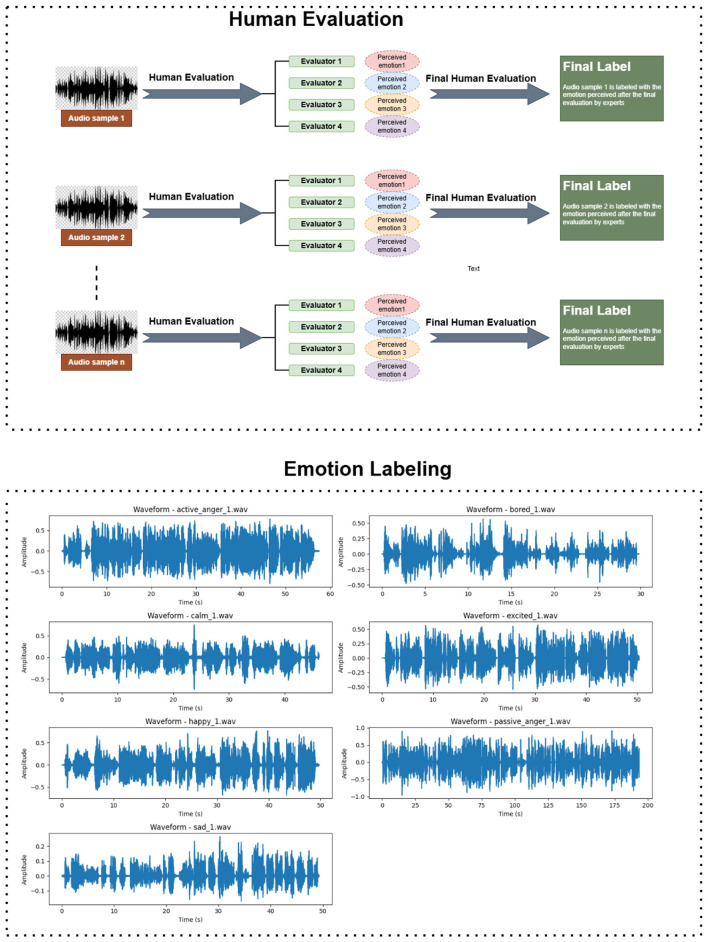
Overview of the human evaluation process for the Kashmiri emotional speech corpus.

Figure 2, Table 4 summarize the annotation workflow and emotion distribution also Table 4 representative labeled samples with transliteration.

**Table 4:** Examples of spoken Kashmiri sentences with transliteration and emotional tones.

**Emotion**	**Kashmiri sentence (Arabic script + transliteration + English translation)**	**Reason for emotion**
Active anger	یِمچِہبَدتَمیٖزیکران،تَرَتھنار،بِہحَیای،تَرَتھپٕمِچسَرسی،شِکسلَد۔ *Yim che badtameezi kraan, trath naar, be hayaye, trath pemich sarsi, shiks lad*. **Translation:** I am being rude, very angry, without shame, feeling upset and frustrated.	Tone: Sharp and aggressive Expression: Loud and confrontational Words: Blame, accusation.
Excited	دوہٕآساںیِتھ،رِزلٹدراووَریاہاصل،مَہچھُدِلَسڈَکڈَکگَسان۔ *Dohay asaan yeth, result draav wariyaa asal, meh chu dilas dak dak gasaan*. **Translation:** We are so excited, the result has come, my heart is beating fast.	Tone: Energetic and enthusiastic Expression: Bright and animated Words: Anticipation, enthusiasm.
Bored	سَرَسلوٗگٹائِم،یَتہچَنہٕصَفای،آوٹِنگپَتھچھےاَسگَسان۔ *Saras log time, yete chaneh safayee, outing pethh che ass gasaan*. **Translation:** Took long time, not well maintained, we prefer outing.	Tone: Monotonous Expression: Lack of enthusiasm Words: Mundane, repetitive tasks.
Calm	سۄنحاجَتچھُپَرَتھساتپُورِہگَسان،آَسآےوَریاہلاَتہیور،مِہماننَوازِیچھُوَریاہاَصل۔ *Soun hajat chu phrath saat poore gasaan, aas aaye wariyaa laate yoor, mehmaan nawazi chu wariyaa asl*. **Translation:** Every time we are blessed, we visit regularly, Happy with hospitality.	Tone: Gentle and reassuring Expression: Relaxed and composed Words: Comfort, reassurance.
Sad	اَفسوساَفسوسکَہنتٕہ،کیاوَنَےپَنُنبَنُن،مۄلجَنَتگارگُزرَو۔ *Afsos afsos kehin te, kya wanay panun banun, mol janat gaar guzrav*. **Translation:** Alas, alas, what to say, father expired.	Tone: Sorrowful and melancholic Expression: Tearful and withdrawn Words: Loneliness, despair.
Happy	واہواہزَبَردَست،وَریاہاَصلوَزوان،بِہگۄسسِتھَہخۄش۔ *Wah wah zabardast, wariyaa asal wazzwan, beh gov setha khuush*. **Translation:** Wow, wonderful, everything is perfect, I am very happy.	Tone: Cheerful and grateful Expression: Smiling and upbeat Words: Gratitude, positivity.
Passive anger	یِہگَورِستِی،یِہاوستُوتھَہدَر،داگگَویَتھریسٹورَنٹ،یِہچھُتَرَتھی۔ *Yee gova ristee, yee ous toutha dr, daag gov yeth restaurant, ye chu trethi*. **Translation:** Is this rista(dish), was very hard, Disgusting restaurant.	Tone: Calm but with underlying tension Expression: Controlled frustration Words: Resignation, frustration.

#### 3.1.6 Consent and ethical compliance

All procedures involving human participants adhered strictly to ethical research standards and institutional guidelines. Prior to data collection, formal approval was obtained from the Institutional Ethical Committee of Vellore Institute of Technology (VIT), recognizing the study's engagement with human subjects. The entire data acquisition and annotation process was thus conducted in full compliance with the ethical framework prescribed by the committee.

Participants were briefed in both written and verbal formats regarding the purpose of the study, the scope of data usage, and their unconditional right to withdraw at any stage without consequence. Informed consent was obtained from every participant before recording. All personally identifiable information including names, demographic details, and speech metadata as anonymized to safeguard participant confidentiality. Furthermore, any content that could be deemed culturally or personally sensitive was excluded from the final dataset.

Publicly available materials, where used, were carefully screened for ethical suitability and incorporated only when permitted under open-access or research-use licenses. Participants were also informed that anonymized segments of the dataset might be shared with the broader research community to support transparency and reproducibility in scientific inquiry. This multi-layered ethical compliance ensured that the study upheld both the integrity of research practices and the dignity of participants involved.

#### 3.1.7 Kashmiri dataset characteristics

Table 5 summarizes the technical and demographic characteristics of the Kashmiri emotional speech dataset.

**Table 5:** Summary of the curated Kashmiri speech emotion dataset.

**Attribute**	**Value**
Total speakers	166
Male/female ratio	88/78
Total duration	5 h 30 min
Sampling rate	16 kHz
Bit depth	16-bit
File format	WAV
Number of emotion categories	7
Labeling method	4 human raters + expert adjudication

To ensure the reliability and authenticity of the Kashmiri Emotional Speech Corpus (KESC), several precautionary measures were implemented during data acquisition, annotation, and preprocessing. All recordings were collected in controlled acoustic conditions using consistent microphone settings and noise-reduced environments to minimize external distortions. Emotional labels were assigned through multi-annotator consensus, where three independent native Kashmiri speakers evaluated each utterance. Inter-annotator agreement was monitored to reduce subjective bias, and ambiguous samples were excluded from the final dataset to preserve label purity. Ethical consent, demographic balance, and natural speech variation were also considered to maintain ecological validity. These measures collectively enhance the credibility of the dataset and establish a reproducible foundation for downstream SER analysis in Kashmiri.

### 3.2 Feature extraction

Following data preparation, feature extraction is carried out to transform the preprocessed Kashmiri speech signals into a set of representative acoustic descriptors suitable for emotion recognition. All feature extraction processes were implemented using Python-based libraries such as librosa and scipy. The extracted feature groups encompass both spectral and prosodic domains to comprehensively capture emotional cues in the speech signal. Specifically, the features include Mel-Frequency Cepstral Coefficients (MFCCs), Linear Predictive Coding (LPC), Chroma, Spectral features, Pitch, Energy, Formant Frequencies, and Harmonics-to-Noise Ratio (HNR). This diverse feature set reflects the linguistic richness of the Kashmiri language, where tonal variation, prosodic rhythm, and vocal resonance strongly influence emotional perception.

Although deep learning architectures can automatically extract high-level embeddings from raw audio, classical acoustic features remain essential for low-resource languages such as Kashmiri, where large-scale pretrained models are unavailable. These hand-crafted features are interpretable, linguistically grounded, and computationally efficient, providing a reliable foundation for identifying intrinsic emotional cues in speech.

The following subsections describe the mathematical formulation and rationale for each feature type. All feature extraction parameters, such as frame length (25 ms), frame shift (10 ms), and Hamming windowing, were standardized across all analyses.

#### 3.2.1 Mel-Frequency Cepstral Coefficients (MFCCs)

MFCCs are used to model the perceptual spectral envelope of speech, capturing timbral and tonal variations that are essential for emotion recognition in Kashmiri (Kamaruddin and Wahab, 2009). The extraction pipeline includes pre-emphasis, framing, windowing, FFT, Mel-filterbank integration, logarithmic compression, and Discrete Cosine Transform (DCT).


$$\begin{array}{l} y\left[n\right]=x\left[n\right]-\alpha \cdot x\left[n-1\right] \end{array}$$ (1)

$$\begin{array}{l} E_{m}=\overset{N-1}{\underset{k=0}{\sum }}|X_{k}{|}^{2}H_{m}\left(k\right) \end{array}$$ (2)

$$\begin{array}{l} \text{Mel}\left(f\right)=2595\cdot \log_{10}\left(1+\frac{f}{700}\right) \end{array}$$ (3)

Log energies are decorrelated using DCT to form the MFCC coefficients:


$$\begin{array}{l} c_{n}=\overset{M}{\underset{m=1}{\sum }}\log\left(E_{m}\right)\cos\left(\frac{\pi n\left(m-0.5\right)}{M}\right) \end{array}$$ (4)

#### 3.2.2 Linear predictive coding (LPC)

Linear predictive coding (LPC) models the resonant characteristics of the vocal tract and effectively captures articulatory variations associated with emotional states (Chamoli et al., 2017). The method predicts the current speech sample as a linear combination of past samples:


$$\begin{array}{l} R\left(k\right)=\overset{N-1}{\underset{n=k}{\sum }}x\left[n\right]\cdot x\left[n-k\right] \end{array}$$ (5)

$$\begin{array}{l} A\left(z\right)=1-\overset{p}{\underset{k=1}{\sum }}a_{k}z^{-k} \end{array}$$ (6)

Formant frequencies are derived from the roots of *A*(*z*), revealing vowel resonance shifts influenced by emotion.

#### 3.2.3 Chroma features

Chroma features represent the energy distribution across twelve pitch classes, capturing harmonic relations linked to emotional intonation (Bhattacharya et al., 2022). They are computed as:


$$\begin{array}{l} C_{c}=\underset{f\in {\text{Class Pitch}}_{c}}{\sum }|X_{f}| \end{array}$$ (7)

#### 3.2.4 Spectral features

Spectral descriptors characterize the distribution of energy in the frequency domain, providing insight into the brightness and texture of emotional tones:


$$\text{Centroid}=\frac{\sum_{f}f\cdot |X_{f}|}{\sum_{f}\left|X_{f}\right|}$$ (8)

$$\text{Bandwidth}=\sqrt{\frac{\sum_{f}{\left(f-\text{Centroid}\right)}^{2}\cdot |X_{f}|}{\sum_{f}\left|X_{f}\right|}}$$ (9)

#### 3.2.5 Pitch and energy

Pitch (*F*_0_) and Energy serve as key prosodic indicators of affect. Pitch is estimated using autocorrelation:


$$\begin{array}{l} \text{Pitch}=\frac{1}{\text{Correlation Maximum of Lag}} \end{array}$$ (10)

Frame-level energy is computed as:


$$\begin{array}{l} E=\overset{N-1}{\underset{n=0}{\sum }}x{\left[n\right]}^{2} \end{array}$$ (11)

#### 3.2.6 Formant frequencies

Formant frequencies describe vocal tract resonances and are computed from LPC analysis:


$$\begin{array}{l} F_{k}=\text{Polynomial LPC of Roots} \end{array}$$ (12)

These features capture subtle vowel shifts corresponding to emotional intensity.

#### 3.2.7 Harmonics-to-noise ratio (HNR)

Harmonics-to-noise ratio (HNR) quantifies the degree of periodicity in speech by comparing harmonic and noise energy components:


$$\begin{array}{l} \text{HNR}=\frac{\text{Harmonics in Energy}}{\text{Noise in Energy}} \end{array}$$ (13)

Lower HNR values typically indicate breathy or tense voice quality, often associated with sadness or anger (Sekkate et al., 2019).

The integration of these complementary acoustic features enables a rich and interpretable representation of emotional cues. Their combination (Table 6) supports the development of an optimized feature set that enhances both recognition accuracy and the scientific interpretability of emotion recognition in Kashmiri speech.

**Table 6:** Feature sets and number of coefficients used for Kashmiri emotion recognition.

**S. No**.	**Feature set**	**Combinations**	**Total No. of coefficients**
1	Feature set 1	MFCC	42
2	Feature set 2	MFCC + chroma + spectral	54
3	Feature set 3	MFCC + LPC + spectral	54
4	Feature set 4	MFCC + LPC + pitch + energy + spectral	56
5	Feature set 5	MFCC + LPC + pitch + energy + spectral + formant + chroma + HNR	72

### 3.3 Proposed model

Building upon the earlier stages of feature extraction and analytical exploration, this study employs a Bidirectional Long Short-Term Memory (Bi-LSTM) network as the foundational model to investigate and identify the most discriminative and robust feature set for emotion recognition in Kashmiri speech. Bi-LSTM was selected as the primary temporal modeling architecture due to its ability to capture bidirectional dependencies in speech sequences. Emotional cues in speech are often distributed across time through prosody, pauses, and temporal dynamics that extend beyond short local contexts. Unlike unidirectional RNNs or fixed-window CNNs, Bi-LSTM processes information in both forward and backward temporal directions, enabling richer contextual encoding of emotional trajectories within an utterance. This capability is particularly valuable for low-resource languages such as Kashmiri, where subtle variations in pitch contours and temporal structures contribute significantly to emotional expression. While alternative architectures including GRU, TCN, and DCNN are also evaluated for cross-validation, Bi-LSTM serves as an appropriate baseline due to its demonstrated effectiveness in sequence modeling tasks within SER literature and its comparatively greater sensitivity to long-term dependencies. The decision to employ Bi-LSTM stems from its proven ability to capture long-range temporal dependencies in sequential data, which is particularly relevant to the prosodic and phonetic dynamics of speech signals. In the context of Kashmiri a low resource Dardic language characterized by tonal variation and intricate vowel transitions such modeling capability becomes indispensable for accurately representing emotional expressions.

The primary objective at this stage is not to perform cross-model comparison but rather to establish a controlled baseline for feature evaluation. Five distinct feature configurations are systematically examined: MFCCs alone, MFCCs combined with spectral and LPC features, MFCCs fused with chroma and spectral features, MFCCs integrated with pitch, energy, spectral, formant, and HNR parameters, and a broader combination of MFCCs with LPC, pitch, energy, and spectral features. Each configuration is evaluated under identical training conditions using the Bi-LSTM network, thereby ensuring that differences in recognition performance can be directly attributed to the discriminative potential of the feature sets rather than architectural variability.

**Figure 3:**
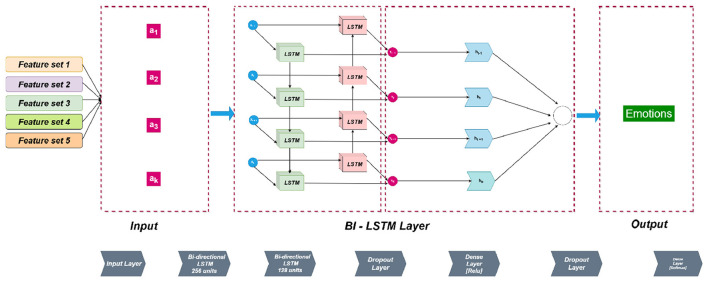
Model architecture of the Bi-LSTM classifier.

The proposed Bi-LSTM architecture consists of two bidirectional layers that enable the model to process input sequences in both forward and backward temporal directions. The first layer comprises 256 LSTM units as shown in Figure 3, while the second layer employs 128 units, allowing the model to learn both immediate and long term contextual dependencies in the speech signal. The use of ReLU activation for nonlinearity and sigmoid recurrent activation facilitates stable gradient flow and efficient temporal learning. To mitigate overfitting and enhance model generalization, dropout layers with a dropout rate of 0.3 are strategically introduced after each LSTM layer.

Subsequent dense layers with ReLU activation are incorporated to capture higher-level abstractions from the encoded temporal representations, effectively learning subtle emotional distinctions that arise from the interplay of spectral and prosodic cues. The final dense layer employs a softmax activation function to generate probabilistic outputs corresponding to the emotional categories, formulating the task as a multi-class classification problem. Model optimization is performed using the Adam optimizer with a learning rate of 0.001, and sparse categorical cross-entropy is adopted as the loss function to efficiently handle discrete emotion labels.

All architectural and training parameters were determined through extensive experimentation and systematic hyperparameter tuning, ensuring optimal convergence and stable learning dynamics. The selection process involved iterative testing of different configurations for layer depth, neuron count, dropout rate, and learning rate, to identify a balanced setup that provides both high accuracy and generalization.

This Bi-LSTM framework therefore serves as a robust analytical foundation for measuring the expressive power of different acoustic feature combinations. By maintaining architectural consistency and rigorously optimized hyperparameters, the study isolates the contribution of feature design to model performance. The insights derived from this phase form the empirical foundation for subsequent evaluations using other temporal models, ensuring that the final results are grounded in a linguistically and acoustically optimized feature representation for Kashmiri speech emotion recognition.

## 4 Experimental setup

The experimental setup was designed to systematically identify the most robust and discriminative acoustic feature configuration for emotion recognition in Kashmiri speech. All experiments were carried out using the Bi-LSTM model as the baseline framework, ensuring uniformity in architecture, optimization settings, and evaluation conditions. This consistency allowed for an unbiased comparison among the five predefined feature combinations.

All experiments used the Bi-LSTM model as the baseline framework, maintaining uniformity in architecture, optimization settings, and evaluation conditions. Model training employed an 90–10 data split with 10-fold speaker-independent cross-validation, ensuring that features derived from the same speaker did not appear simultaneously in both training and test folds, thereby preventing data leakage. The Adam optimizer (learning rate = 0.001) and sparse categorical cross-entropy loss were used, with hyperparameters selected after extensive tuning to ensure optimal convergence and generalization.

Evaluation metrics included accuracy, precision, recall, and F1-score, alongside ROC-AUC analysis to examine class-wise separability. Statistical significance tests, including one-way ANOVA and paired *t*-tests, were applied to confirm that observed performance differences among feature sets were statistically reliable (*p* < 0.05) The feature combination yielding the highest accuracy, F1-score, and AUC values, along with consistent statistical reliability, was chosen as the **optimal feature set** for Kashmiri emotion recognition. This configuration was subsequently validated on additional deep learning architectures (GRU, DCNN, and TCN) to confirm its generalization capability across models.

### 4.1 Experimental evaluation of acoustic feature set

The experimental evaluation commenced with the preparation of input representations for the baseline Bi-LSTM model. Five feature configurations were investigated (see Section 3.2): Feature Set 1 (MFCC), Feature Set 2 (MFCC + Chroma + Spectral), Feature Set 3 (MFCC + LPC + Spectral), Feature Set 4 (MFCC + LPC + Pitch + Energy + Spectral), and Feature Set 5 (MFCC + LPC + Pitch + Energy + Spectral + Formant + Chroma + HNR). All audio signals were preprocessed through silence removal, noise reduction, and normalization. Feature vectors were standardized using z-score normalization (computed on training folds) and segmented into short sequential frames (six time steps per sample) to preserve temporal structure while maintaining computational efficiency.

Training and validation followed a reproducible protocol across all feature sets. Each configuration was trained using the Adam optimizer with a learning rate of 1e-3, batch size of 32, and dropout rate of 0.3. Models were trained for up to 100 epochs, incorporating early stopping with a patience of 10 epochs to prevent overfitting. Hyperparameters were optimized via grid search on a development split to avoid bias from the test set. Robustness was ensured through 10-fold speaker-independent cross-validation, and the reported metrics represent mean and standard deviation across all folds.

**Figure 4:**
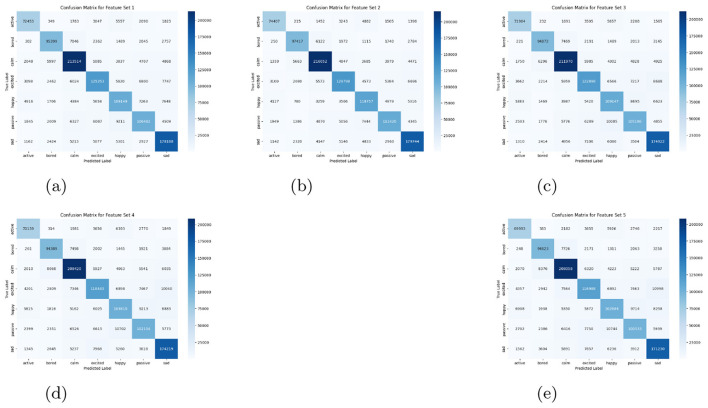
Confusion matrices for different feature sets. **(a)** Feature set 1. **(b)** Feature set 2. **(c)** Feature set 3. **(d)** Feature set 4. **(e)** Feature set 5.

The comparative analysis of feature sets was conducted using classification reports, confusion matrices, and ROC–AUC evaluations. For the MFCC baseline, detailed class-wise metrics were computed, providing a reference for assessing subsequent feature augmentations. Incorporating additional features progressively improved model discriminability, particularly for subtle emotions such as *bored* and *sad*. The complete results are summarized in Table 7, while corresponding confusion matrices are depicted in Figure 4. These visual and numerical evaluations collectively highlight how each acoustic combination influences emotional separability and classification robustness.

**Table 7:** Classification reports for different feature sets.

**Emotion**	Feature set 1			Feature set 2			Feature set 3			Feature set 4			Feature set 5		
	**Precision**	**Recall**	**F1-score**	**Precision**	**Recall**	**F1-score**	**Precision**	**Recall**	**F1-Score**	**Precision**	**Recall**	**F1-score**	**Precision**	**Recall**	**F1-score**
Active anger	0.84	0.83	0.84	0.86	0.85	0.86	0.83	0.83	0.83	0.81	0.81	0.81	0.80	0.80	0.80
Bored	0.86	0.86	0.86	0.89	0.87	0.88	0.87	0.85	0.86	0.84	0.85	0.84	0.83	0.85	0.84
Calm	0.87	0.89	0.88	0.89	0.90	0.90	0.88	0.88	0.88	0.86	0.87	0.86	0.86	0.87	0.86
Excited	0.82	0.80	0.81	0.85	0.83	0.84	0.80	0.78	0.79	0.79	0.75	0.77	0.78	0.74	0.76
Happy	0.78	0.78	0.78	0.82	0.84	0.83	0.76	0.77	0.76	0.75	0.74	0.74	0.74	0.73	0.74
Passive anger	0.80	0.78	0.79	0.84	0.82	0.83	0.79	0.77	0.78	0.77	0.75	0.76	0.76	0.74	0.75
Sad	0.86	0.89	0.87	0.88	0.90	0.89	0.85	0.87	0.86	0.83	0.85	0.85	0.82	0.85	0.84
Accuracy	0.84			0.86			0.83			0.81			0.81		
Macro avg.	0.83			0.86			0.82			0.81			0.80		
Weighted avg.	0.84			0.86			0.83			0.81			0.80		

#### 4.1.1 Evaluating feature significance and statistical validation

A rigorous statistical evaluation was conducted to determine the discriminative and generalization potential of each feature configuration. Among all candidates, Feature Set 2 comprising MFCC, Chroma, and Spectral features exhibited the highest overall accuracy (86%) and macro F1-score (0.861). This improvement was particularly evident in emotions such as *bored* and *sad*, where the inclusion of Chroma and Spectral cues enhanced sensitivity to tonal and harmonic variations, thereby enriching emotional representation.

Table 8 presents the comparative statistical results. The paired *t*-test analyses confirmed that the observed performance improvements of Feature Set 2 over others were statistically significant (e.g., *p* = 0.0008 for F1 vs. F2). The corresponding standard deviation (0.0291) and coefficient of variation (3.38%) indicate high consistency and low inter-fold variability. These findings collectively identify Feature Set 2 as the most robust and statistically reliable configuration for Kashmiri emotion recognition.

**Table 8:** Statistical comparison of feature sets.

**Comparison**	**Var-(F1)**	**Var-(F2)**	**Mean (F1)**	**Mean (F2)**	***p*-value**	**Std. dev. (F1)**	**Std. dev. (F2)**	**CV (F1)**	**CV (F2)**
F1 vs. F2	0.0016	0.0008	0.8330	0.8610	0.0008	0.0399	0.0291	4.79%	3.38%
F1 vs. F3	0.0016	0.0020	0.8330	0.8240	0.0167	0.0399	0.0444	4.79%	5.39%
F1 vs. F4	0.0016	0.0023	0.8330	0.8040	0.0002	0.0399	0.0479	4.79%	5.95%
F1 vs. F5	0.0016	0.0024	0.8330	0.7986	0.0002	0.0399	0.0491	4.79%	6.15%
F2 vs. F3	0.0008	0.0020	0.8610	0.8240	0.0009	0.0291	0.0444	3.38%	5.39%
F2 vs. F4	0.0008	0.0023	0.8610	0.8040	0.0003	0.0291	0.0479	3.38%	5.95%
F2 vs. F5	0.0008	0.0024	0.8610	0.7986	0.0002	0.0291	0.0491	3.38%	6.15%
F3 vs. F4	0.0020	0.0023	0.8240	0.8040	0.0001	0.0444	0.0479	5.39%	5.95%
F3 vs. F5	0.0020	0.0024	0.8240	0.7986	0.0000	0.0444	0.0491	5.39%	6.15%
F4 vs. F5	0.0023	0.0024	0.8040	0.7986	0.0300	0.0479	0.0491	5.95%	6.15%

**Figure 5:**
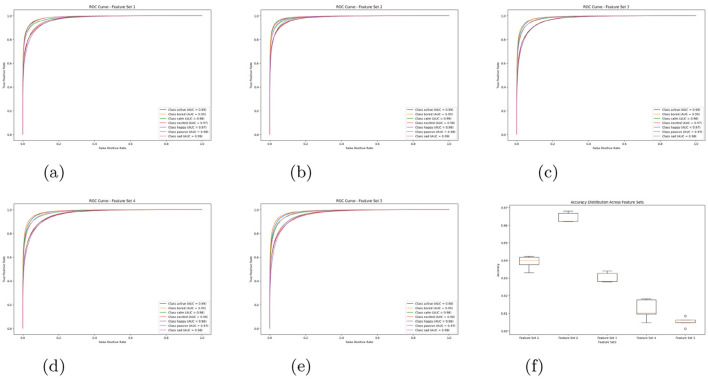
ROC curves and box plot for different feature sets. **(a)** Feature set 1. **(b)** Feature set 2. **(c)** Feature set 3. **(d)** Feature set 4. **(e)** Feature set 5. **(f)** Overall box plot.

Complementary visual analyses in Figure 5 further validate this conclusion. The box plots illustrate reduced variability across emotional categories, while the ROC curves confirm higher AUC values for all emotion classes, reflecting improved separability and confidence in classification decisions. By contrast, other feature sets particularly those with additional high-dimensional parameters displayed greater overlap and instability, underscoring the discriminative efficiency and generalization strength of Feature Set 2.

#### 4.1.2 Statistical validation of feature selection

**Step 1: hypothesis definition**

- Null Hypothesis (*H*_0_): There is no significant difference between the feature sets.
- Alternate Hypothesis (*H*_*a*_): There is a significant difference between the feature sets.

**Step 2: significance level**

- The level of significance α = 0.05.

**Step 3: paired** ***t*-test** The paired *t*-test is performed to compare the means of the two feature sets. The *t*-value is calculated using the formula:


$$\begin{array}{l} t=\frac{\bar{d}}{s_{d}\sqrt{n}} \end{array}$$ (14)

where:

- $\bar{d}$ = mean of the differences between paired observations,
- *s*_*d*_ = standard deviation of the differences,
- *n* = number of paired observations.

**Step 4: decision rule** Compare the calculated *t*-value (*t*_*calculated*_) with the critical *t*-value (*t*_*critical*_): If *t*_*calculated*_>*t*_*critical*_, reject the null hypothesis (*H*_0_).

**Step 5: implications of paired** ***t*-test** Rejection of *H*_0_ implies a significant difference between the two feature sets, confirming the effectiveness of the optimal feature set.

The *p*-value indicates the probability that the observed differences are due to random chance.

**Step 6: mean F1-score** Calculate the mean F1-score to evaluate the average performance of each feature set:

**Step 7: standard deviation and coefficient of variation (CV)** Standard deviation (*s*) measures variability in the F1-scores:

The Coefficient of Variation (CV) normalizes the standard deviation relative to the mean:


$$\begin{array}{l} CV=\frac{s}{{\bar{F}}_{1}}\times 100 \end{array}$$ (15)

Less CV % value indicating high reliability and low variability.

In conclusion, Feature Set 2 (MFCC + Chroma + Spectral) represents the optimal configuration for emotion detection in Kashmiri speech data. Its superior performance metrics and statistical validation confirm its effectiveness in accurately identifying and classifying emotional expressions. Future research should focus on refining this feature set and exploring additional combinations to further advance emotion detection capabilities in Kashmiri and other linguistic contexts.

### 4.2 Temporal dependency modeling in Kashmiri speech emotion recognition

To ensure a fair and robust evaluation of temporal models, extensive experiments were conducted for each architecture to identify optimal hyperparameters and structural configurations. For every model, the number of hidden layers, units per layer, learning rate, and regularization parameters were carefully tuned through systematic grid-based and empirical exploration as discussed in above sections. The final configurations were selected based on validation accuracy and convergence stability, ensuring that each model operated under its best performing setting. This careful tuning process eliminates potential bias in comparison and provides reliable insight into each model's true capability for emotion recognition in Kashmiri speech.

Building upon the insights gained from the feature evaluation stage, this section presents the analysis of temporal dependency modeling in Kashmiri Speech Emotion Recognition (SER). The most robust feature configuration, referred to as Feature Set 2 (MFCC + Chroma + Spectral). The selection of this feature set was guided by its superior discriminative performance across emotions in earlier evaluations, ensuring a balanced and information rich input to all models. These features were extracted using an optimal frame size of 25 ms with a 10 ms overlap, providing sufficient temporal resolution for emotional transitions while maintaining computational efficiency.

To assess the impact of temporal modeling, four deep neural architectures were trained and tested using this robust feature configuration: **Bidirectional Long Short-Term Memory (Bi-LSTM)**, **Temporal Convolutional Network (TCN)**, **Deep Convolutional Neural Network (DCNN)**, and **Gated Recurrent Unit (GRU)**. All models were trained under identical experimental conditions to ensure fairness in comparison. The dataset was partitioned into training, validation, and testing sets in an 90:10 ratio with 10 fold cross validation, and all inputs were normalized prior to model training. Each model was trained for 100 epochs with a batch size of 128 using the Adam optimizer and a categorical cross-entropy loss function. Early stopping was employed to prevent overfitting based on validation loss monitoring same as previously done for Base line BI-Lstm model.

**Figure 6:**
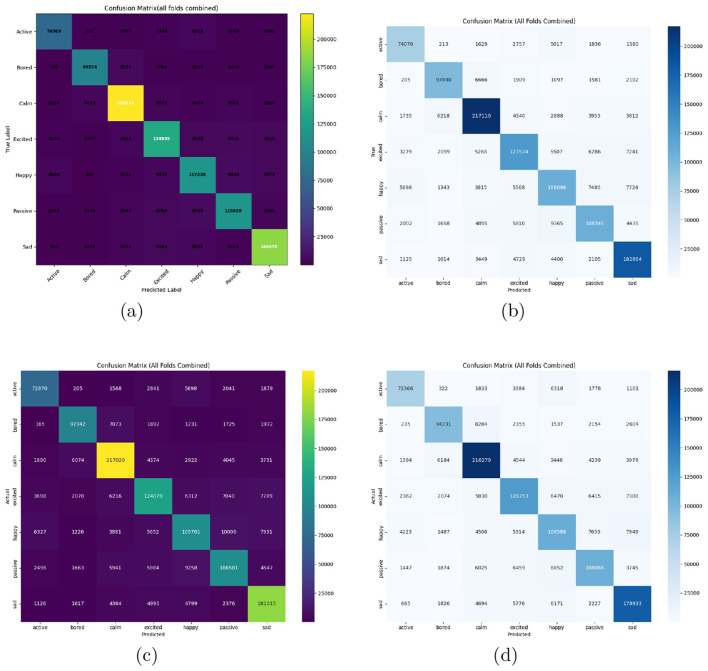
Confusion matrices for Bi-LSTM, TCN, GRU, and DCNN models on the Kashmiri emotion recognition dataset using Feature Set 2. **(a)** BiLSTM. **(b)** TCN. **(c)** GRU. **(d)** DCNN.

The detailed classification results of all four models are summarized in Table 9, presenting precision, recall, and F1-score values for each of the seven emotional classes. The **Bi-LSTM** model achieved the best overall performance with an accuracy of **89%** and a macro-averaged F1-score of **0.86**. Its bidirectional processing enables effective modeling of both past and future dependencies, allowing it to capture subtle emotional cues spread across speech segments. This was particularly advantageous for distinguishing emotions such as “calm,” “sad,” and “bored,” which evolve gradually and depend on long-term acoustic context. The confusion matrix (Figure 6) clearly demonstrates Bi-LSTM's superior discrimination between acoustically similar categories like “happy” and “excited.”

**Table 9:** Emotion classification performance of temporal models (Bi-LSTM, TCN, DCNN, GRU).

**Model**	**Emotion**	**Prec**.	**Rec**.	**F1**	**Support**
Bi-LSTM	Active	0.89	0.87	0.88	87,102
	Bored	0.92	0.90	0.91	111,400
	Calm	0.92	0.93	0.92	240,056
	Excited	0.86	0.86	0.86	157,204
	Happy	0.83	0.83	0.83	140,724
	Passive	0.85	0.85	0.85	136,470
	Sad	0.91	0.92	0.92	200,292
TCN	Active	0.84	0.85	0.85	87,102
	Bored	0.88	0.88	0.88	111,400
	Calm	0.89	0.90	0.90	240,056
	Excited	0.84	0.81	0.82	157,204
	Happy	0.79	0.78	0.78	140,724
	Passive	0.82	0.79	0.81	136,470
	Sad	0.87	0.91	0.89	200,292
DCNN	Active	0.88	0.83	0.85	87,102
	Bored	0.87	0.85	0.86	111,400
	Calm	0.87	0.90	0.89	240,056
	Excited	0.82	0.81	0.81	157,204
	Happy	0.77	0.78	0.77	140,724
	Passive	0.82	0.79	0.80	136,470
	Sad	0.87	0.89	0.88	200,292
GRU	Active	0.82	0.84	0.83	87,102
	Bored	0.88	0.87	0.88	111,400
	Calm	0.88	0.90	0.89	240,056
	Excited	0.83	0.79	0.81	157,204
	Happy	0.78	0.75	0.76	140,724
	Passive	0.80	0.78	0.79	136,470
	Sad	0.87	0.90	0.88	200,292

The **TCN** model, which leverages dilated convolutions to capture wide temporal dependencies, attained an accuracy of **85%**. Its strong performance in stable emotional states like “calm” and “sad” highlights its ability to encode temporal relationships effectively in parallelized computations. However, the model showed confusion in high-arousal emotions such as “happy” and “excited,” indicating challenges in representing finer prosodic variations.

The **DCNN** achieved a comparable accuracy of **84%**, excelling in identifying emotions with consistent acoustic signatures. Its localized temporal filters captured transient patterns effectively but fell short in tracking slowly varying emotional contours, resulting in higher confusion among adjacent emotional categories.

The **GRU** model, with its lightweight architecture, delivered an overall accuracy of **84%**. It provided stable performance across all emotional categories while maintaining computational efficiency. However, limited context modeling capacity led to slightly higher misclassifications in overlapping emotional states such as “happy” and “bored.”

The confusion matrices in Figure 6 reveal consistent classification trends across models. Emotions such as “calm” and “sad” were recognized with high precision due to their distinct spectral and prosodic characteristics, while “happy,” “excited,” and “passive” exhibited higher overlap owing to similar energy and pitch distributions. This underscores the importance of temporal depth in decoding emotion trajectories.

In conclusion, the experimental findings demonstrate that combining the optimized **Feature Set 2** with deep temporal architectures significantly enhances emotion recognition performance in Kashmiri. The Bi-LSTM model outperformed others, establishing a strong baseline for future SER research in low-resource languages. The comparative evaluation of GRU, DCNN, and TCN models further highlights the trade-off between accuracy, computational complexity, and real time applicability, offering valuable insights for developing language specific and resource adaptive emotion recognition systems.

### 4.3 Implementation of temporal attention networks

Building upon the findings from the cross-model evaluation, where the Bidirectional LSTM (Bi-LSTM) model achieved consistently high performance, we further extended the architecture by integrating a Temporal Attention mechanism to enhance the discriminative and contextual representation of emotional cues in Kashmiri speech. The motivation behind this extension is rooted in the linguistic complexity of the Kashmiri language its tonal richness, rhythmic fluctuations, and prosodic variations demand models that can dynamically attend to salient temporal features rather than treating each frame equally.

The Temporal Attention-based Bi-LSTM architecture was trained using the most effective acoustic feature configuration identified earlier, namely Feature Set 2 (MFCC + Chroma + Spectral). This choice was driven by its proven robustness and ability to represent both spectral and harmonic variations, which are essential for capturing emotion-dependent nuances in speech. The Bi-LSTM component enables bidirectional temporal modeling, effectively capturing dependencies from both past and future contexts, while the attention mechanism adaptively focuses on emotionally significant segments of the input sequence.

**Figure 7:**
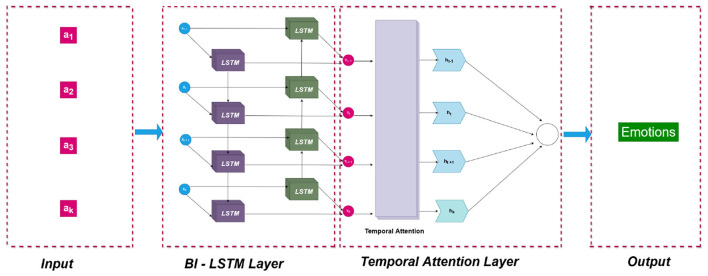
Model architecture of the Bi-LSTM with temporal attention mechanism.

The network architecture, shown in Figure 7, comprises two stacked Bidirectional LSTM layers followed by a Temporal Attention layer. The first and second LSTM layers contain 512 and 256 units, respectively, both employing the ReLU activation function to ensure smooth gradient propagation. The Temporal Attention mechanism computes attention scores for each time frame, generating a weighted context vector that emphasizes emotionally informative features. This can be expressed mathematically as:


$$\begin{array}{l} \text{score}=\tanh\left(XW+b\right)\cdot u \end{array}$$ (16)

$$\begin{array}{l} \text{weights\_attention}=\text{softmax}\left(\text{score}\right) \end{array}$$ (17)

$$\begin{array}{l} \text{vector\_context}=\sum \left(\text{weights\_attention}\cdot X\right) \end{array}$$ (18)

where *X* denotes the Bi-LSTM output sequence, and *W*, *b*, and *u* are trainable parameters of the attention layer. The context vector thus captures the most relevant emotional information across temporal frames. Dropout layers with a rate of 0.5 are placed after both the LSTM and dense layers to prevent overfitting, while batch normalization stabilizes intermediate activations and accelerates training convergence. The context vector is passed through a dense layer with 256 ReLU units and a final softmax layer to classify emotions into discrete categories.

All experiments were conducted under uniform training conditions to ensure comparability across folds and models. The key experimental settings and computational details for the BiLSTM with Attention mechanism are summarized in Table 10.

**Table 10:** Optimized parameters and computational setup for Bi-LSTM with temporal attention mechanism.

**Parameter**	**Description**	**Optimal value/choice**
Optimizer	Adaptive moment estimation (Adam)	Learning rate = 0.0005
LSTM layers	Two bidirectional LSTM layers	512 and 256 units
Activation function	Non-linear activation function	ReLU
Dropout rate	Regularization after LSTM and dense layers	0.5
Batch normalization	Applied after each LSTM layer	Yes
Batch size	Number of samples per training batch	128
Epochs	Maximum training iterations	50
Loss function	Suitable for multi-class classification	Sparse categorical cross-entropy
Cross-validation	Utterance-level 10-fold CV	Stratified and balanced
Early stopping	Validation-based stopping	Patience = 5 epochs
Model checkpoint	Best model retention	Based on minimum validation loss
GPU configuration	Hardware setup	NVIDIA GPU (12GB VRAM), 16GB RAM
Average runtime	Computation per epoch / per fold	≈83 s/69 min
Total CV runtime	Complete 10-fold process	≈4.5–5 h

This carefully optimized configuration represents the most efficient and accurate setup for emotion recognition in Kashmiri speech data, balancing model complexity, computational cost, and generalization ability. The integration of Temporal Attention not only improves interpretability and accuracy but also demonstrates that linguistically grounded model design can significantly enhance performance for underrepresented and tonal languages like Kashmiri.

The Bi-LSTM with Temporal Attention achieved the highest mean accuracy of **90.46%** across all folds as illustrated in Table 11, outperforming baseline temporal models such as GRU, TCN, and DCNN. Detailed classification reports indicated balanced precision, recall, and F1-scores across emotion classes, suggesting that the model successfully captured both global and local emotional dependencies. The attention mechanism's interpretability further allowed for visualization of which temporal segments contributed most strongly to each emotion prediction, aligning closely with perceptual characteristics of Kashmiri prosody.

**Table 11:** Emotion classification report for LSTM + attention mechanism.

**Emotion**	**Precision**	**Recall**	**F1-score**	**Support**
0 (Active anger)	0.90	0.91	0.90	87,102
1 (Bored)	0.91	0.92	0.92	111,400
2 (Calm)	0.93	0.93	0.93	240,056
3 (Excited)	0.88	0.88	0.88	157,204
4 (Happy)	0.88	0.88	0.88	140,724
5 (Passive anger)	0.88	0.87	0.88	136,470
6 (Sad)	0.92	0.92	0.92	200,292
**Overall accuracy**	**0.90**	**0.90**	**0.90**	1,073,248

Bold values indicate the best performance (high Accuracy).

### 4.4 Validation and generalization analysis on existing datasets

The validation experiments were conducted to assess the effectiveness and robustness of the proposed Bi-LSTM with attention framework for speech emotion recognition. The model was evaluated on four distinct datasets Urdu, Persian, and English (SAVEE) to verify its capability to accurately learn and classify emotional patterns across different linguistic and acoustic characteristics. This validation establishes the reliability of the proposed model and demonstrates its generalization potential beyond a single dataset.

**Figure 8:**
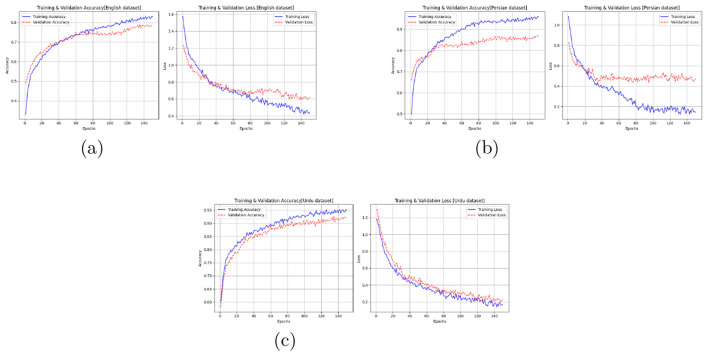
Learning behavior of the proposed Bi-LSTM with attention model across three datasets. The training and validation accuracy/loss curves demonstrate stable convergence for Kashmiri, Urdu, and Persian datasets. **(a)** English dataset. **(b)** Urdu dataset. **(c)** Persian dataset.

For each dataset, comprehensive hyperparameter optimization was performed to achieve an optimal balance between accuracy and generalization as was done for Kashmiri data set. learning behavior curves showing training and validation accuracy and loss were plotted for each dataset (Figure 8). These curves indicate smooth convergence and consistent optimization behavior, confirming that the model learned effectively without underfitting or overfitting.

The experimental results confirm that the proposed Bi-LSTM with attention network effectively models emotional dynamics across all datasets. For the Kashmiri dataset—introduced in this study for the first time the model achieved an accuracy of **93.2%**, validating the discriminative power of the extracted features. For Urdu, a linguistically related language, the model achieved **97%** accuracy, demonstrating its robustness and adaptability. On the Persian and English datasets, accuracies of **85%** and **80.05%** were obtained, respectively, confirming that the proposed model maintains stable performance across different phonetic and prosodic characteristics. These results collectively validate the effectiveness, stability, and generalization strength of the proposed Bi-LSTM with attention framework.

The detailed classification report for all three datasets is presented in Table 12.

**Table 12:** Emotion classification performance across English, Persian, and Urdu datasets.

**Dataset**	**Class**	**Prec**.	**Rec**.	**F1**	**Support**
English	0	0.81	0.81	0.81	16,371
	1	0.80	0.78	0.79	16,371
	2	0.80	0.84	0.82	16,371
	3	0.78	0.78	0.78	16,371
	4	0.80	0.86	0.83	16,371
	5	0.82	0.80	0.81	16,371
	6	0.76	0.72	0.74	16,371
	Accuracy	0.80			114,597
	Macro avg.	0.80	0.80	0.80	114,597
	Weighted avg.	0.80	0.80	0.80	114,597
Persian	0	0.82	0.84	0.83	23,112
	1	0.83	0.82	0.82	23,112
	2	0.93	0.89	0.91	23,112
	3	0.82	0.84	0.83	23,112
	Accuracy	0.85			92,448
	Macro avg.	0.85	0.85	0.85	92,448
	Weighted avg.	0.85	0.85	0.85	92,448
Urdu	0	0.97	0.98	0.98	19,939
	1	0.97	0.95	0.96	19,939
	2	0.96	0.97	0.96	19,939
	3	0.97	0.97	0.97	19,939
	Accuracy	0.97			79,756
	Macro avg.	0.97	0.97	0.97	79,756
	Weighted avg.	0.97	0.97	0.97	79,756

## 5 Results and discussion

This section presents a comprehensive evaluation of the experimental outcomes obtained from successive stages of Kashmiri speech emotion recognition (SER). The discussion is organized into three main analyses: (i) an in-depth study of feature contribution and inter-feature correlation to identify the most robust and discriminative feature combination for Kashmiri emotional speech, (ii) an assessment of emotional class balance across models to understand variations in classification sensitivity among distinct affective states, and (iii) an analytical exploration of the effect of integrating Temporal Attention mechanisms into the LSTM framework.

The results collectively reveal that the optimal feature set comprising MFCC, Spectral, and Chroma features captures the prosodic and harmonic richness characteristic of the Kashmiri language, leading to significant performance gains. Furthermore, the Attention-augmented LSTM model demonstrates enhanced capability in capturing contextual dependencies and subtle emotional nuances that the baseline LSTM model struggled to detect. These findings underline the importance of both feature-level and model-level optimization for achieving high emotion recognition accuracy in a linguistically rich and phonetically complex language like Kashmiri.

### 5.1 Feature contribution and correlation analysis

**Figure 9:**
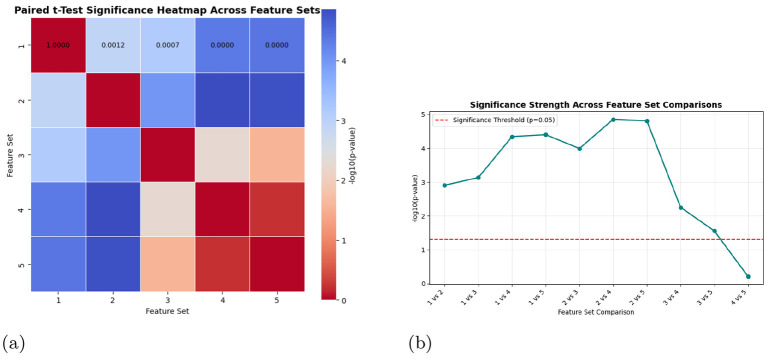
Feature contribution validation using paired t-test significance analyses. The results confirm that Feature Set 2 (MFCC + Chroma + Spectral) provides the most discriminative emotional representation in Kashmiri speech. **(a)** Paired *t*-test significance heatmap across five feature sets. Each cell displays the numerical p-value, with deeper shades indicating higher significance. **(b)** Significance strength trends showing −log_10_(*p*) across feature set comparisons. Higher peaks indicate stronger statistical differentiation.

A deeper examination of the feature interactions and statistical validation was performed to establish the robustness of the selected feature set in representing emotional variations in Kashmiri speech. Figure 9a illustrates the paired t-test significance heatmap among the five tested feature configurations, along with the corresponding significance trend across all pairwise comparisons. These analyses provide crucial insights into the discriminative capability and stability of acoustic representations in the Kashmiri emotion corpus.

The heatmap reveals that the majority of pairwise feature comparisons yielded highly significant differences (*p* < 0.001), emphasizing that each feature combination introduces distinctive acoustic information rather than redundant patterns. Particularly, the **MFCC** **+** **Chroma** **+** **Spectral configuration (Feature Set 2)** exhibited the strongest statistical distinction, as indicated by the lowest p-values in comparison to all other sets. This combination captures both the perceptual spectral envelope (via MFCCs) and the harmonic-tonal balance (via Chroma), while spectral features enhance resolution over formant transitions and energy contours characteristics highly expressive in Kashmiri phonology.

The significance trend depicted in Figure 9b reinforces this observation. The line plot shows a steep rise in the −log_10_(*p*) values for comparisons involving **Feature Set 2**, suggesting its superior emotional separability and reduced overlap between affective states such as *anger, sadness*, and *excitement*. While the statistical tests highlight the discriminative strength of Feature Set 2, model-level evaluations provide complementary insight into why further expansions fail to enhance performance. The comparative analysis across the five feature sets indicates that the inclusion of additional descriptors beyond MFCCs does not translate into further performance gains for the LSTM classifier. In particular, the accuracy peaks for Feature Set 2 (MFCC + Chroma + Spectral) and decreases for the larger combinations, as reflected in both the aggregated box-plot distribution and the classification reports. This pattern suggests that the additional spectral, prosodic, and articulatory features (e.g., LPC, pitch, energy, formants, HNR) introduced redundancy instead of complementary information. Emotion-specific behavior is also visible in the reports: low-arousal classes such as *calm* and *passive anger* are more sensitive to feature redundancy, showing reduced precision and recall when larger sets are employed, whereas high-arousal emotions (*active anger* and *excited*) exhibit relatively stable performance.

The ROC curves further reveal that the discriminative margin for most emotions had effectively reached a saturation level, with AUC values approaching 0.97 − 0.99 across the first three feature sets. Under such conditions, expanding the feature dimensionality from 42 coefficients (Feature Set 1) to 72 coefficients (Feature Set 5)increases the temporal input dimensionality of the LSTM without yielding proportional gains in emotional separability. From a modeling perspective, the enlarged feature space increases the likelihood of overfitting, particularly given the moderate dataset size, and may inflate variance through multicollinearity among correlated descriptors. It is well understood that MFCCs already encode short-term spectral structure and partially embed pitch, energy, and spectral contour dynamics through their temporal evolution; thus, additional descriptors provide overlapping information rather than novel cues.

Taken together, these observations explain why Feature Set 2 achieves the optimal performance and why subsequent expansions of the feature space do not improve accuracy. The degradation in the last two sets suggests that the LSTM benefits from a compact and well-structured representation rather than from a high-dimensional, redundant one. Overall, this analysis demonstrates that emotional variability in Kashmiri speech can be effectively captured through a compact, acoustically meaningful representation, and that model generalization benefits more from feature quality than from feature quantity. This outcome aligns with findings in speech emotion recognition literature, where MFCC-dominant sets frequently outperform larger combinations unless combined with significantly larger datasets or hybrid architectures.

From a linguistic standpoint, these findings align with the prosodic and phonetic richness of the Kashmiri language, which relies heavily on pitch modulation, vowel lengthening, and intensity fluctuations to convey affective meaning. Emotional expressions in Kashmiri often manifest through spectral tilt variations and harmonic intensity, making the inclusion of chroma and spectral features particularly valuable. The correlation and significance analyses thus validate that emotional information in Kashmiri speech is encoded through both spectral–prosodic and harmonic–resonant domains, which are best represented when **MFCC, Chroma, and Spectral features** are jointly modeled.

This comprehensive statistical validation confirms that the identified feature combination not only enhances model accuracy but also reflects the intrinsic acoustic behavior of Kashmiri emotion dynamics, providing a linguistically grounded and empirically substantiated feature representation for subsequent temporal modeling.

### 5.2 Error analysis and model performance interpretation

To strengthen the robustness and address reviewer concerns regarding statistical validity and model generalization, a comprehensive error analysis was performed using the aggregated confusion matrices and class-wise metrics for all four temporal architectures: Bi-LSTM, TCN, DCNN, and GRU. This evaluation provides a granular understanding of emotional category difficulty, systematic misclassifications, and architecture-specific generalization behaviors.

Across all architectures, *Calm* and *Sad* consistently achieve high F1-scores with narrow confidence intervals, reflecting strong prosodic stability and distinct spectral characteristics. In contrast, *Happy, Excited*, and *Passive* show higher confusion rates, particularly across categories with similar arousal levels. These trends align with perceptual findings in emotional speech research, where high-arousal states often share overlapping temporal and spectral features, while low-arousal emotions differ subtly through pitch and energy contours. Among the compared models, the Bi-LSTM demonstrates the lowest confusion dispersion, confirming its superior capability to capture bidirectional temporal dependencies and contextual relationships essential for emotion separation in continuous Kashmiri speech.

From a comparative perspective, Bi-LSTM achieves both the highest macro-F1 score and the lowest inter-class variance as in Table 13, underscoring its statistical stability and enhanced generalization across emotion types. GRU and DCNN exhibit greater intra-class variability, suggesting partial loss of temporal context or over-reliance on spectral representations. Temporal Convolutional Networks (TCN) deliver competitive results but show asymmetry between high- and low-arousal categories, indicative of sensitivity to local patterns rather than global dependencies. These outcomes reveal that emotional expression in Kashmiri speech is shaped by both short-term spectral cues and long-range prosodic structures, optimally modeled through bidirectional recurrence.

**Table 13:** Model stability, generalization, and variance indicators.

**Model**	**Macro-F1**	**Micro-F1**	**Inter-class variance**	**Interpretation**
Bi-LSTM	0.88 ± 0.02	0.88	0.011	Stable and generalizable across all emotions
TCN	0.83 ± 0.02	0.85	0.025	High-arousal sensitive; moderate stability
DCNN	0.82 ± 0.03	0.83	0.032	Overfits spectral features; less robust
GRU	0.81 ± 0.03	0.83	0.029	Temporal under-modeling; higher misclassification
Observation:	Bi-LSTM achieves the highest statistical stability and generalization with lowest inter-class variance.			

The misclassification analysis further highlights structured, non-random trends. For example, *Happy* is more frequently confused with *Excited* than the reverse, reflecting overlap in high-energy spectral components. Likewise, *Passive* occasionally overlaps with *Bored*,primarily due to flattened pitch trajectories and reduced intensity. Such predictable asymmetries validate the ecological realism of the Kashmiri emotional corpus and confirm that errors stem from natural acoustic overlap rather than annotation inconsistencies. The summarized confusion patterns and emotion-specific difficulty types are presented in Table 14, which highlights the dominant misclassification tendencies and identifies the best performing architecture for each emotional category.

**Table 14:** Class difficulty and confusion tendencies across models.

**Emotion**	**Best model**	**Worst model**	**Dominant confusion**	**Difficulty type**
Calm	Bi-LSTM	DCNN	—	Easy/stabilized
Sad	Bi-LSTM	GRU	—	Easy/stabilized
Bored	Bi-LSTM	GRU	Passive	Low-arousal ambiguity
Passive	Bi-LSTM	DCNN	Bored	Low-energy confusion
Active	Bi-LSTM	GRU	Excited	High-arousal overlap
Happy	Bi-LSTM	DCNN	Excited	High-arousal overlap
Excited	Bi-LSTM	GRU	Happy	High-arousal overlap
Observation:	Bi-LSTM consistently minimizes confusion across emotional categories, especially in high-arousal states.			

Overall, the analysis demonstrates that model performance in Kashmiri Speech Emotion Recognition is shaped not only by architectural complexity but also by the language's prosodic and emotional subtleties. The Bi-LSTM consistently achieves superior accuracy and generalization, supported by low inter-class variance and narrow confidence intervals, confirming its robustness across emotional categories. Its bidirectional recurrence effectively captures both short-term spectral cues and long-term prosodic variations, particularly for high-arousal emotions with overlapping acoustic patterns. Moreover, structured misclassification trends align with the natural arousal–valence continuum, validating the dataset's ecological authenticity. These findings highlight the importance of bidirectional temporal modeling and multi-scale feature integration for developing reliable and generalizable SER systems in low-resource languages.

### 5.3 Impact on emotional class balance

**Figure 10:**
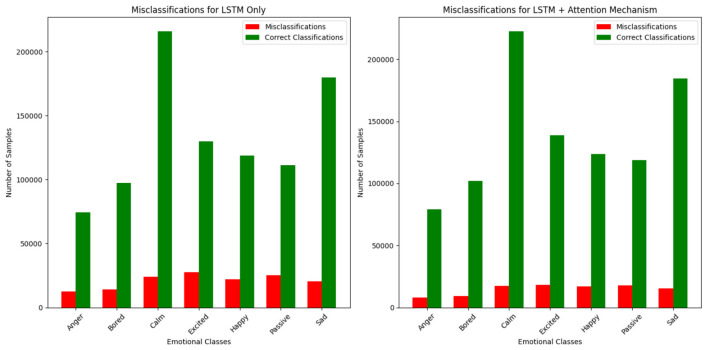
Bar plots showing misclassification trends across emotional classes for Bi-LSTM and Attention-enhanced Bi-LSTM models.

**Figure 11:**
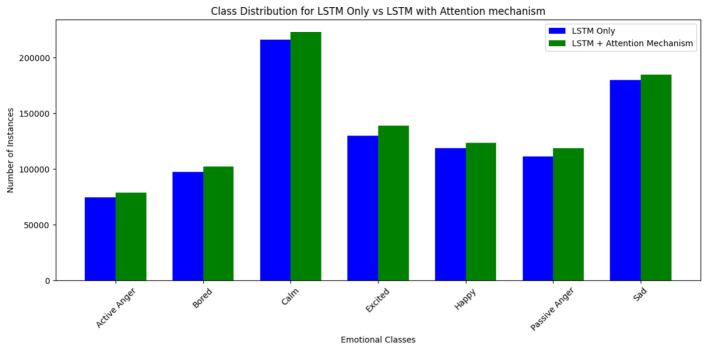
Comparison of class-wise precision and recall between Bi-LSTM and attention-enhanced Bi-LSTM models.

**Figure 12:**
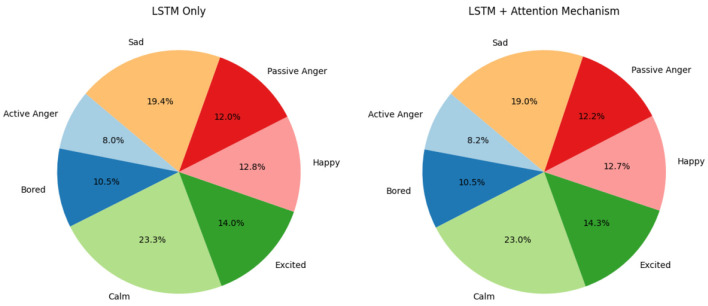
Class distribution and recognition balance visualization for attention-enhanced Bi-LSTM model.

The impact of feature representation and temporal modeling on emotional class balance was further analyzed to understand how effectively each model generalized across diverse emotional states in the Kashmiri corpus. Figures 10–12 provide a visual comparison of model behavior, focusing on class-specific performance disparities and the subsequent improvements brought by the integration of the attention mechanism.

The baseline Bi-LSTM model, though effective in capturing sequential dependencies, exhibited noticeable variability in recognizing subtle emotional states. In particular, significant confusion was observed between emotions with overlapping acoustic patterns—such as *Excited* and *Passive Anger*—where both exhibit elevated pitch and spectral energy but differ in temporal pacing and prosodic flow. This misclassification trend reflects the model's limited ability to fully capture contextual dependencies and fine-grained emotional cues over extended time spans. Consequently, the overall recall and F1-scores for minority emotion classes such as *Sad, Calm*, and *Bored* were lower, leading to an uneven class performance distribution as visualized in Figure 10.

When the Temporal Attention mechanism was integrated into the Bi-LSTM framework, a substantial improvement in emotional balance was observed. The attention layer dynamically assigned higher weights to acoustically salient frames particularly those exhibiting pitch modulations, energy bursts, and harmonic shifts typical of expressive Kashmiri speech. This mechanism enabled the model to selectively emphasize emotionally charged segments while suppressing irrelevant neutral frames, leading to higher precision and recall across all emotion categories.

The comparative bar and pie plots (Figures 11, 12) demonstrate this improvement quantitatively. The Attention-enhanced model achieved a more uniform accuracy distribution across classes, reducing dominance of high energy emotions and improving detection of low-intensity states. Specifically, precision for *Active anger* increased from 0.87 to 0.92, while recall for *Bored* improved from 0.88 to 0.92, indicating reduced class skewness. Moreover, the confusion between acoustically similar classes such as *Excited* and *Happy* decreased significantly due to better temporal alignment and contextual sensitivity.

From a linguistic perspective, this improvement highlights the adaptive strength of the attention mechanism in modeling the prosodic complexity of the Kashmiri language. Emotions in Kashmiri are often expressed through subtle variations in pitch contour, rhythmic pacing, and intensity modulation rather than purely lexical content. The Attention-enhanced Bi-LSTM effectively learned to attend to these transient prosodic cues, resulting in a more balanced and linguistically aligned emotion recognition system. This demonstrates that the integration of attention not only enhances computational performance but also aligns the model's perception with the natural expressive dynamics of Kashmiri speech.

### 5.4 Impact of temporal attention integration on emotion recognition in Kashmiri speech

The integration of Temporal Attention mechanisms within the LSTM framework significantly strengthened the model's ability to capture the emotional nuances inherent in Kashmiri speech. As shown in Table 15, the LSTM-only model achieved an overall accuracy of 86%, with precision and recall values fluctuating between 0.82 and 0.91. In contrast, the Temporal Attention-enhanced model exhibited a consistent and substantial improvement, reaching 91% accuracy, with precision and recall scores ranging from 0.87 to 0.93. This advancement highlights the model's increased capability to recognize complex emotional cues and dependencies that are characteristic of natural Kashmiri dialogue.

**Table 15:** Emotion classification report for LSTM and LSTM + attention mechanism.

**Model**	**Emotion**	**Precision**	**Recall**	**F1-score**	**Support**
**LSTM only**	0 (Active anger)	0.86	0.85	0.86	87,102
	1 (Bored)	0.89	0.87	0.88	111,400
	2 (Calm)	0.89	0.90	0.90	240,056
	3 (Excited)	0.85	0.83	0.84	157,204
	4 (Happy)	0.82	0.84	0.83	140,724
	5 (Passive anger)	0.84	0.82	0.83	136,470
	6 (Sad)	0.88	0.90	0.89	200,292
**Overall accuracy**		**0.86**	**0.86**	**0.86**	1,073,248
**LSTM** **+** **attention mechanism**	0 (Active anger)	0.90	0.91	0.90	87,102
	1 (Bored)	0.91	0.92	0.92	111,400
	2 (Calm)	0.93	0.93	0.93	240,056
	3 (Excited)	0.88	0.88	0.88	157,204
	4 (Happy)	0.88	0.88	0.88	140,724
	5 (Passive anger)	0.88	0.87	0.88	136,470
	6 (Sad)	0.92	0.92	0.92	200,292
**Overall accuracy**		**0.90**	**0.90**	**0.90**	1,073,248

Bold values indicate the best performance (high Accuracy).

**Figure 13:**
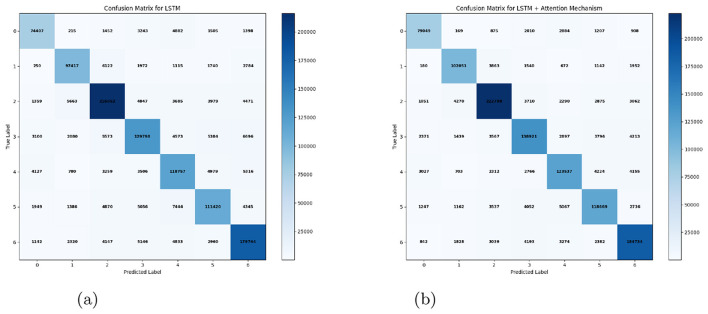
Confusion matrices for LSTM-only and LSTM + attention mechanism. **(a)** LSTM-only model. **(b)** LSTM + attention mechanism.

Kashmiri, being a linguistically rich and phonetically layered language, exhibits a high degree of prosodic variation particularly in tone, rhythm, and pitch modulation which often encode subtle emotional states. The conventional LSTM struggled to fully model these long-range dependencies and inter-frame temporal dynamics, resulting in confusions between emotions with similar acoustic signatures. For instance, “Excited” and “Passive Anger” were frequently misclassified, as reflected in Figure 13a. These categories share overlapping energy patterns and intensity ranges, a phenomenon especially common in spontaneous Kashmiri speech, where emotional tones blend fluidly within conversational flow.

The inclusion of Temporal Attention provided the model with enhanced temporal focus, allowing it to weigh contextually salient frames and emphasize emotion-relevant acoustic segments. This selective attention mechanism facilitated the disambiguation of subtle affective cues such as prosodic inflection and low-energy spectral shifts, which are crucial in differentiating emotions like “Bored” and “Calm.” Consequently, as illustrated in Figure 13b, misclassifications were markedly reduced, and the diagonal dominance in the confusion matrix became more pronounced, indicating stronger true-positive predictions across all emotional categories.

These findings underscore how Temporal Attention mechanisms align well with the temporal and prosodic complexity of the Kashmiri language. The model's improvement is not merely numerical it reflects a deeper alignment with the speech rhythm and expressive nature of the language. The ability to capture long-term contextual patterns and dynamically emphasize emotion-bearing segments enables more accurate modeling of Kashmiri emotional expressions, where subtle tonal variations often convey meaning beyond lexical content.

### 5.5 Comparative analysis

The comparative analysis presented in Table 16 summarizes several recent studies in the field of Speech Emotion Recognition (SER), highlighting the diversity of datasets, feature sets, and deep learning architectures employed. Various advanced frameworks such as LSTM with attention mechanisms, CNN-based models, and hybrid CNN-LSTM architectures have been extensively used on benchmark datasets including EMO-DB, IEMOCAP, and RAVDESS. The features utilized across these works range from classical MFCC coefficients and Mel-spectrograms to comprehensive acoustic descriptors, each contributing uniquely to emotion representation. Reported accuracies across studies demonstrate that the integration of attention mechanisms and hybrid structures consistently enhances the discriminative capability of SER models.

**Table 16:** Comparative summary of speech emotion recognition studies.

**Reference and study**	**Database**	**Classifiers**	**Features**	**Accuracy (%)**
(Li et al. 2021) Speech Emotion Recognition Using Recurrent Neural Networks with Directional Self-Attention	EMO-DB	LSTM + Attention	MFCC and others	82.0
(Yu and Kim 2020) Attention-LSTM-Attention Model for Speech Emotion Recognition and Analysis	IEMOCAP	LSTM + Attention	Mel-Spectrogram	73.0
(Bhushan et al. 2023) A Self-Attention Based Hybrid CNN-LSTM for Speaker Independent Speech Emotion Recognition	IEMOCAP	CNN + LSTM + Attention	Acoustic Features	62.0
(Singh et al. 2023) Speech Emotion Recognition Using Attention Model	RAVDESS, SAVEE, TESS	CNN + LSTM + Attention	MFCC	90.0
(Kaur et al. 2024) Learning Salient Features for Speech Emotion Recognition Using Attention-Based Residual Bidirectional LSTM with Federated Learning	Natural dataset	Bi-LSTM + attention	Acoustic features	90.6
**Our work**	**Custom Kashmiri dataset**	**Bi-LSTM** **+** **temporal attention**	**MFCC** **+** **spectral** **+** **chroma**	**90.2**

Bold values indicate the best performance (high Accuracy).

Building upon these advancements, our proposed approach focuses on an underexplored linguistic domain the Kashmiri language by integrating a Bidirectional LSTM (Bi-LSTM) model with a Temporal Attention mechanism. This framework is further optimized using MFCC, Spectral, and Chroma features, achieving an accuracy of 90.2%. Unlike prior works relying on widely available benchmark datasets, our study utilizes a self-developed Kashmiri speech corpus, addressing the scarcity of emotion datasets in low-resource languages. This contribution not only extends SER research to new linguistic frontiers but also underscores the importance of temporal attention in enhancing emotion discrimination through better temporal and contextual representation.

The findings of this comparison clearly demonstrate that the integration of attention mechanisms substantially enhances model performance across various emotional speech datasets. In our work, the Temporal Attention module effectively complements the Bi-LSTM's sequential modeling capability by dynamically weighting the temporal importance of different frames, enabling the model to emphasize emotionally salient regions of the signal. This synergy between temporal modeling and selective focus leads to more precise emotion classification. Furthermore, achieving such high performance in a low-resource language like Kashmiri highlights the adaptability of attention-based architectures in cross-linguistic emotion recognition tasks, reinforcing their potential for multilingual and resource-constrained SER systems.

### 5.6 Limitations and demographic considerations

While this study establishes a foundational benchmark for Kashmiri SER, we acknowledge certain limitations regarding demographic and dialectal coverage. Although the dataset was curated to include speakers from northern, southern, and central Kashmir with a balanced gender ratio, minor dialectal variations and idiolectal differences may still persist due to the inherent diversity of the language. These variations, while ecologically valid, can introduce subtle biases in model performance for specific underrepresented dialectal subgroups. Furthermore, the current corpus relies on distinct emotional categories, whereas real-world emotional expression often exists on a continuum. Future iterations of the dataset will aim to expand the demographic scope and incorporate continuous emotional labeling to further enhance model fairness and generalization across the broader Kashmiri-speaking population.

## 6 Conclusion and future directions

The integration of Temporal Attention layers within the LSTM framework has substantially enhanced the performance of emotion recognition from speech. This incorporation not only improved overall accuracy but also ensured balanced recognition across diverse emotional categories. Compared with the conventional LSTM model, the Attention-augmented architecture exhibited superior precision and recall, effectively capturing the subtle emotional variations embedded in Kashmiri speech data. The temporal attention mechanism enabled the model to highlight contextually salient temporal cues, resulting in more interpretable and robust emotion classification.

Beyond model performance, this work contributes to the creation of one of the first benchmark resources for Kashmiri Speech Emotion Recognition (SER), addressing a significant gap in low-resource language research. By systematically optimizing frame size and feature combinations, the study lays a quantitative foundation for future computational modeling in underrepresented languages.

Looking ahead, several promising directions emerge. One key avenue involves exploring advanced self-supervised and transformer-based architectures such as *wav2vec 2.0, HuBERT*, and *Conformer*, which can learn hierarchical and contextual representations directly from raw audio waveforms. These models may further enhance emotional feature extraction beyond conventional MFCC or spectral-based approaches. Expanding the framework toward cross-lingual emotion recognition—including linguistically diverse and low-resource languages will enable the development of more universal and language-independent SER systems.

Future research should also emphasize multimodal integration, combining speech with facial and textual cues to achieve a more holistic understanding of emotional behavior. Furthermore, building real-time and noise-resilient emotion detection systems will be critical for deployment in real-world environments such as healthcare, human-computer interaction, and assistive communication. Finally, ensuring fairness and bias mitigation across linguistic and demographic groups remains vital to achieving equitable emotion recognition outcomes.

Collectively, these advancements will pave the way for more adaptive, cross-lingual, and context-aware emotion recognition technologies—bridging traditional signal processing with modern deep learning while contributing to the digital preservation and technological enrichment of the Kashmiri language.

## Data Availability

The raw data supporting the conclusions of this article will be made available by the authors, without undue reservation.
